# Case Report: A case of post-viral inflammatory insomnia: observed sleep restoration associated with histamine-targeted interventions and implications for mast cell pathways

**DOI:** 10.3389/frsle.2025.1736866

**Published:** 2026-01-20

**Authors:** Amanda Jill Meckes, James William Meckes

**Affiliations:** 1 RestoreWellnessRx, Baton Rouge, LA, United States; 2 Sheffield, AL, United States

**Keywords:** dysautonomia, histamine, inflammation, inflammatory insomnia, mast cells, post-viral syndrome, sleep regulation

## Abstract

**Background:**

Mast cell-mediated inflammation has been proposed as a potential contributor to neuroinflammatory insomnia and dysautonomia, but objective clinical documentation remains limited. Histamine and related immune mediators can disrupt circadian rhythm, arousal systems, and autonomic stability. Conventional pharmacological therapies for sleep restoration often fail to address these inflammatory mechanisms.

**Case presentation:**

A previously healthy and active 74-year-old male presented with post-viral dysautonomia and severe, treatment-refractory insomnia accompanied by persistent sneezing and ocular irritation suggestive of histamine reactivity. Despite optimal CPAP use and multiple pharmacological trials for sleep disturbance (zolpidem, trazodone, gabapentin, diazepam, lemborexant), Oura Ring data demonstrated persistently low sleep scores, often in the 30–40 range, and minimal REM and deep sleep. Routine laboratory studies (CBC, CMP, thyroid, cortisol, testosterone) were normal except for mildly low DHEA-S, consistent with chronic inflammatory stress. Following evaluation by a tertiary sleep specialist who suggested daytime stimulant therapy (declined by the patient), a targeted supportive regimen was initiated to promote physiologic recovery and restore sleep architecture. This included phosphatidylcholine, alpha-GPC, coenzyme Q10, cyproheptadine (2–4 mg at qHS) and removal of scented household products. Sleep metrics improved within 24 h, with Oura sleep scores increasing to 75+ from a 10-month period characterized by nightly scores often in the 30–40 range and remained stable thereafter. At 4-week follow-up, lingering daytime fatigue and patient-reported cognitive fog prompted additional dietary modification to a low-histamine pattern and the addition of loratadine (10 mg AM) and famotidine (20 mg BID).

**Outcome:**

Within several days, the patient reported marked improvement in energy, cognition, and overall functional capacity, following nearly a year of functional incapacitation.

**Conclusion:**

This case demonstrates a reversible form of inflammatory insomnia and fatigue, likely mediated by mast cell-driven histamine activity. A multi-component intervention targeting histamine pathways, including antihistamine therapy, environmental modification, and dietary adjustment, was associated with rapid and sustained normalization of objective sleep metrics in this patient. These findings highlight the importance of evaluating immune and inflammatory contributors in patients with refractory insomnia and support further investigation of mast cell-related pathways in translational sleep medicine.

## 1 Introduction

Mast cell–mediated inflammation has been increasingly recognized as a potential contributor to neurovascular, immune, and autonomic dysregulation. Aberrant mast-cell activation results in excess release of histamine, cytokines, and other mediators that extend beyond classical allergic pathways to influence neural signaling and sleep regulation. Histamine, a potent wake-promoting neurotransmitter, activates neurons in the hypothalamic tuberomammillary nucleus and can disrupt normal sleep architecture when persistently elevated (Scammell et al., 2019; Yoshikawa et al., 2020; Venner et al., 2019). Emerging evidence suggests that immune-mediated inflammation may contribute to chronic insomnia and dysautonomia through this pathway. Patients with such presentations often exhibit postural intolerance, cognitive impairment, and fatigue; symptoms frequently misattributed to primary psychiatric or sleep disorders. For the purposes of this report, *inflammatory insomnia* refers to sleep disruption in which immune-mediated signaling is hypothesized to play a primary mechanistic role.

We present this case to describe the clinical phenotype, document the temporal association of a multi-component, histamine-targeted intervention with objective and subjective improvement, and propose mast cell–mediated inflammation as a testable hypothesis for a subset of patients with post-viral, refractory insomnia and dysautonomia (Huang et al., 2024; Nishino et al., 2022; Nakao and Nakamura, 2022).

## 2 Case presentation

### 2.1 Diagnostic assessment and clinical context

At presentation, the patient's clinical phenotype suggested an immune-mediated process characterized by post-viral onset, histamine-type symptoms (persistent sneezing, ocular irritation), dysautonomia, and severe sleep disruption. Formal diagnostic testing for mast cell activation syndrome (MCAS), including acute-phase serum tryptase or urinary histamine metabolites, was not obtained, representing a limitation of this case (Weiler, 2020). Alternative causes of refractory insomnia were considered, including untreated sleep apnea, medication-induced insomnia, primary psychiatric insomnia, and neurodegenerative disease. These were felt to be less likely given excellent CPAP adherence and efficacy, minimal response to multiple sedative-hypnotic trials, preserved cognition prior to the post-viral illness, and the absence of prior psychiatric history. Diagnostic reasoning therefore favored a post-viral inflammatory and histamine-mediated mechanism as the parsimonious explanation for the patient's symptom constellation.

The patient, a 74-year-old male who was previously healthy and active, presented with post-viral fatigue, dysautonomia, persistent sneezing and ocular irritation, and profound insomnia. Despite excellent CPAP adherence and documented therapeutic efficacy, Oura Ring data demonstrated chronically low sleep scores, often in the 30–40 range, with minimal REM and deep sleep. In activities of daily living, he was functionally incapacitated, spending more than 20 h per day supine. Trials of zolpidem, trazodone, gabapentin, lemborexant, and diazepam failed to improve sleep quality or daytime energy. A university-based sleep specialist was unable to determine the cause of the refractory insomnia, confirming adequate CPAP efficacy. Stimulant therapy for daytime alertness was offered and declined by the patient (Chen et al., 2023; Kadl et al., 2025).

Comprehensive cardiac evaluation and routine laboratory testing (CBC, CMP, thyroid function, cortisol, testosterone) were within normal limits, with the exception of mildy reduced DHEA-S, a finding that has been reported in chronic inflammatory states and may reflect impaired adrenal reserve in the setting of persistent immune activation. The constellation of profound fatigue, allergic symptoms, and autonomic instability suggested a prolonged state of cellular and mitochondrial stress. To support physiologic recovery, adjunctive therapy was initiated with phosphatidylcholine, alpha-GPC, and coenzyme Q10, targeting cellular membrane integrity, acetylcholine synthesis, and mitochondrial function, while concurrently initiating cyproheptadine (2 mg qHS) to modulate histamine activity (National Institute of Diabetes Digestive Kidney Diseases, 2017; Bertrand et al., 2021). While antihistamine therapy appeared to be the principal driver of symptomatic improvement, concurrent cellular and mitochondrial support may have facilitated more rapid physiologic recovery by optimizing membrane stability and acetylcholine signaling.

Environmental modifications were also implemented, including removal of indoor volatile organic compounds (VOCs) through elimination of plug-in air fresheners, discontinuation of scented detergent and toiletries, and removal of indoor floral arrangements (Potera, 2011; Steinemann, 2016). Sleep metrics improved within 24 h, with Oura Ring sleep scores rising from a baseline period characterized by scores often in the 30–40 range to 75 overnight, following approximately 10 months of sleep fragmentation (Svensson et al., 2024).

At 4-week follow-up, the patient reported sustained improvement in sleep but persistent daytime fatigue, subjective cognitive sluggishness, and difficulty with recent central weight gain. He also noted bloating and reflux associated with specific foods. A low-histamine dietary pattern was introduced, eliminating common dietary histamine triggers, along with initiation of loratadine (10 mg each morning) and famotidine (20 mg twice daily; Kou et al., 2024). Within several days, the patient experienced a marked increase in energy and reported improvement in cognition. A detailed sequence of interventions and outcomes is summarized in Table 1.

**Table 1:** Timeline of clinical events and interventions.

**Date/period**	**Clinical events and interventions**	**Outcome/ observations**
Month 0–10	Post-viral dysautonomia with profound insomnia. Oura Ring sleep scores often in the 30–40 range, with minimal REM and deep sleep. Multiple medication trials failed (zolpidem, trazodone, gabapentin, diazepam, lemborexant).	Functional incapacitation; 20 h/day supine; severe fatigue and subjective cognitive decline.
Month 10	Evaluation confirmed normal CPAP function and unremarkable labs except mildly reduced DHEA-S. Initiated supportive regimen: phosphatidylcholine, alpha-GPC, and CoQ10. Environmental modifications included removal of plug-in air fresheners, scented detergents, and floral arrangements. Began cyproheptadine 2–4 mg qHS.	Sleep restored overnight (Oura score ≈ 75+). REM and deep sleep normalized.
Month 11	Lingering daytime fatigue prompted low-histamine diet plus loratadine 10 mg AM and famotidine 20 mg BID.	Rapid increase in energy, subjective cognitive clarity, and daytime function.
Month 11 + 1 week onward	Maintained low-histamine diet and antihistamine regimen.	Sustained sleep stability and functional recovery.

**Figure 1:**
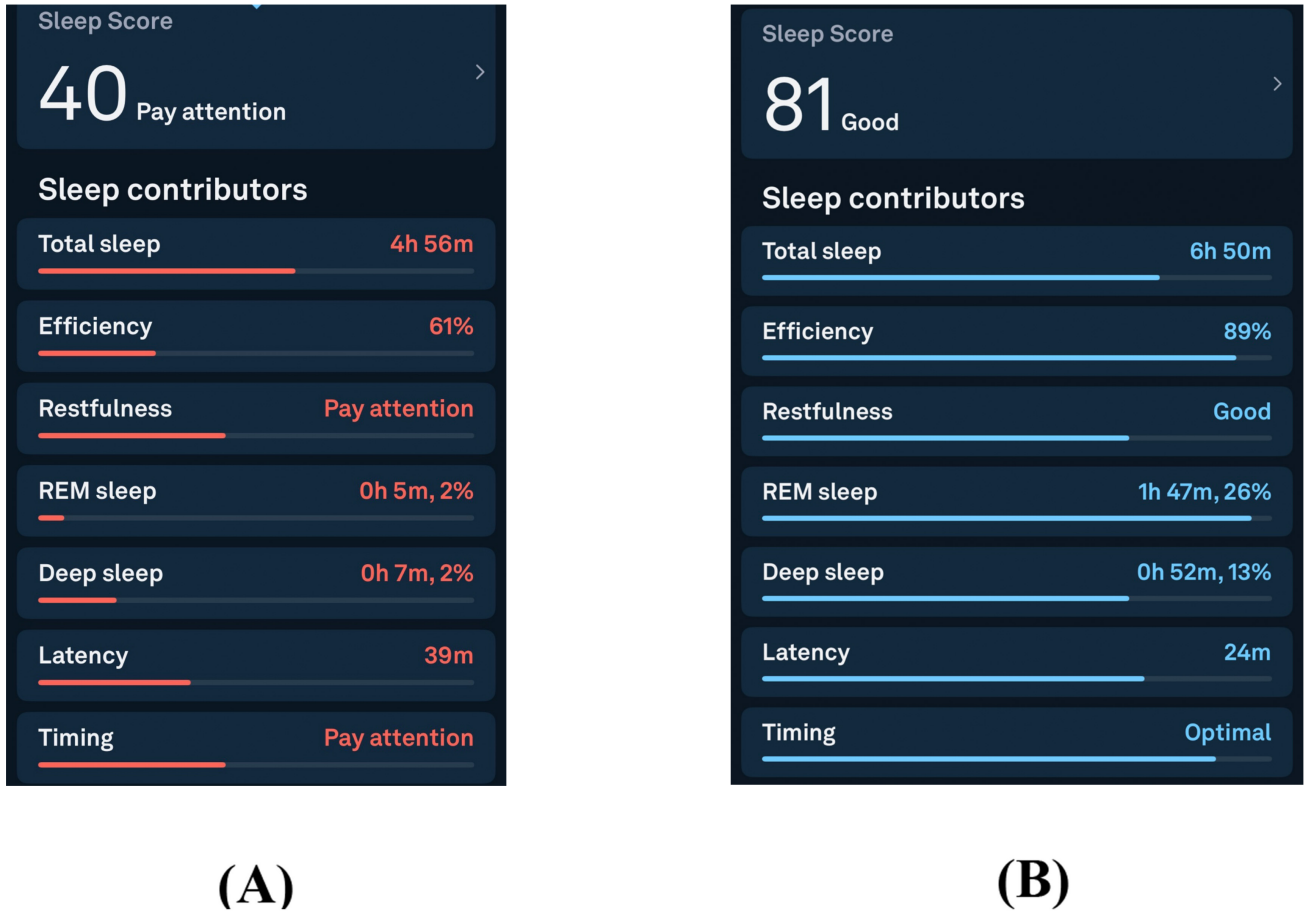
Oura Ring sleep metrics before **(A)** and after **(B)** targeted antihistamine and environmental interventions. Panel A reflects the approximately 10-month pre-intervention period preceding July 2025, during which sleep scores were persistently low, often in the 30–40 range, with minimal REM and deep sleep. Panel B reflects the early post-intervention period beginning in July 2025, demonstrating rapid improvement in wearable-estimated total sleep time, sleep efficiency, REM, and deep sleep duration. Metrics were derived using the Oura Ring Generation 3 proprietary sleep staging algorithm, which has demonstrated validity relative to ambulatory polysomnography but does not provide direct EEG measurement (Svensson et al., 2024). Improvements followed initiation of a multi-component antihistamine-targeted strategy (Kou et al., 2024).

**Figure 2:**
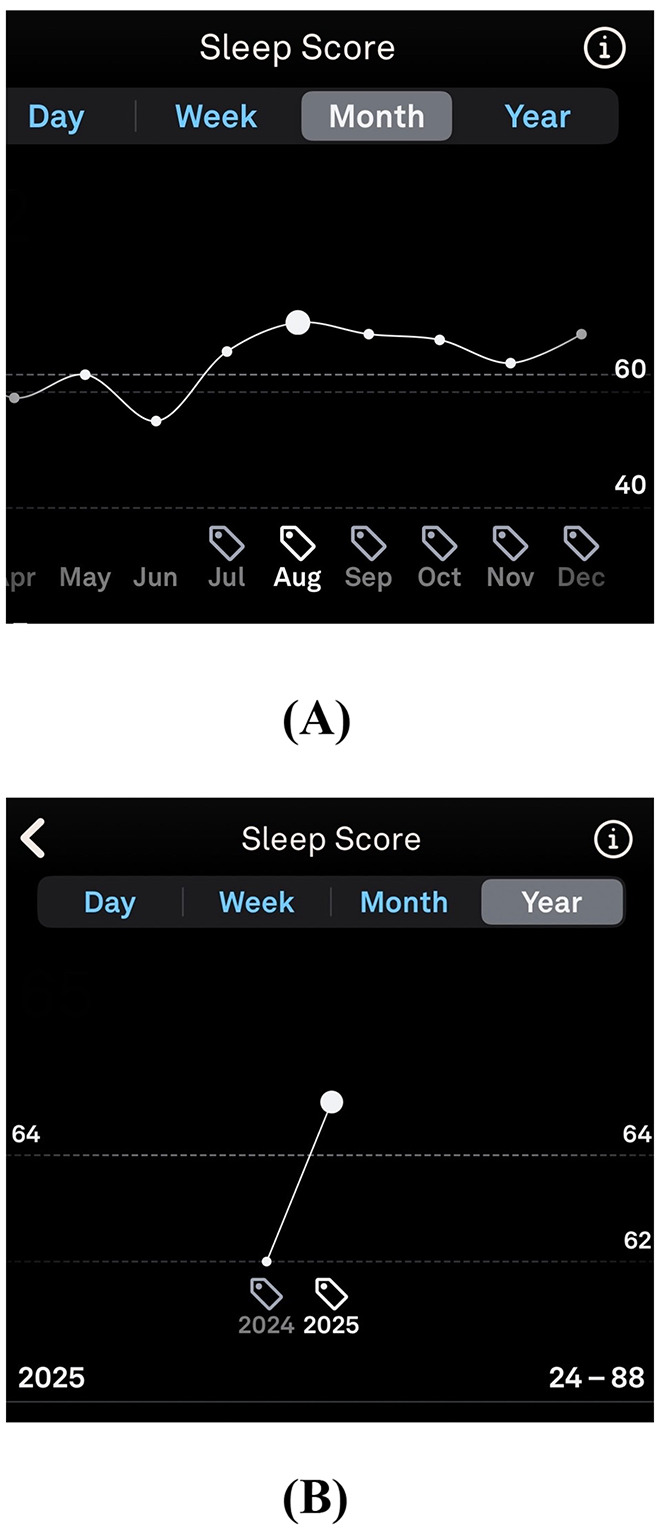
Longitudinal Oura Ring-derived sleep score trends before and after intervention. **(A)** Year-level sleep score trends demonstrate persistently low sleep scores during the pre-intervention period, with nadir values reaching the mid-20s, followed by marked improvement after initiation of the targeted antihistamine and environmental intervention strategy. **(B)** Month-level sleep score trends illustrate sustained improvement and stabilization of sleep metrics across subsequent months following intervention. Sleep scores were derived from the Oura Ring Generation 3 proprietary algorithm and reflect wearable-estimated sleep quality rather than direct EEG-measured sleep architecture.

See Figure 1 for Oura Ring-derived improvements in total sleep duration, sleep efficiency, REM sleep estimates, and estimated deep sleep following intervention. Longitudinal wearable data further demonstrated a marked and sustained improvement in sleep metrics following intervention (Figure 2).

## 3 Discussion

This case describes a temporal association between a multi-component, histamine-targeted intervention strategy and objective improvement in sleep metrics in a patient with post-viral, treatment-refractory insomnia. The rapid and sustained improvement in device-verified sleep metrics following antihistamine therapy, cellular support, dietary modification, and environmental trigger removal is suggestive of a reversible immune-mediated contribution to sleep disruption in this patient. Sleep metrics improved within 24 h, with Oura Ring sleep scores rising from a baseline period characterized by sleep scores often in the 30–40 range to 75 overnight, accompanied by increases in wearable-estimated REM and deep sleep duration. As wearable sleep staging relies on algorithmic inference rather than direct EEG measurement, these changes should be viewed as estimates of sleep architecture rather than definitive markers of physiologically normal, homeostatically regulated slow wave sleep. These findings generate a testable hypothesis that mast cell–mediated inflammation may contribute to a subset of refractory insomnia cases, particularly in individuals with concurrent allergic and dysautonomic symptoms (Huang et al., 2024; Nishino et al., 2022; Nakao and Nakamura, 2022).

As a theoretical framework to explain the observed symptom constellation, chronic mast cell activation may secondarily influence hypothalamic–pituitary–adrenal (HPA) axis function through persistent inflammatory signaling. Cytokine-mediated suppression of adrenal output and mitochondrial stress could contribute to reductions in cortisol and DHEA, thereby amplifying fatigue, stress intolerance, autonomic instability, and central adiposity. The patient's mildly reduced DHEA-S level and central weight gain are consistent with, but not diagnostic of, this downstream effect. Additionally, gastrointestinal symptoms including bloating and reflux may reflect histamine-mediated enteric nervous system activation and altered vagal signaling. This proposed neuroimmune–endocrine model remains speculative and requires validation in controlled studies (Huang et al., 2024; Nishino et al., 2022; Nakao and Nakamura, 2022).

From a systems perspective, it is plausible that immune activation, neuroendocrine suppression, and metabolic dysregulation may form a self-reinforcing cycle in susceptible individuals, with histamine pathways playing a contributory role. In this case, the bundled intervention of histamine modulation, membrane and mitochondrial support, and elimination of environmental and dietary triggers was temporally associated with marked functional recovery. However, the simultaneous initiation of multiple interventions precludes attribution of effect to any single component, and improvement may reflect synergistic, additive, placebo, or natural recovery effects rather than direct mechanistic reversal.

## 4 Strengths and limitations

Strengths of this report include the use of prospective, objective sleep tracking with longitudinal wearable data, detailed clinical phenotyping, and extended follow-up documenting sustained improvement. However, several important limitations must be emphasized. First, this is a single-case observation and therefore cannot establish causality or generalizability. Second, formal biochemical confirmation of mast cell activation (e.g., serial serum tryptase, urinary histamine or prostaglandin D_2_ metabolites) was not obtained, leaving the proposed mast cell mechanism unverified. Third, the bundled nature of the intervention, including antihistamine therapy, supplements, dietary modification, and environmental control, introduces significant confounding. In particular, cyproheptadine possesses intrinsic sedative and antiserotonergic effects that alone could influence sleep initiation independent of histamine modulation. Fourth, the absence of blinding, randomization, and control for the natural history of post-viral recovery introduces susceptibility to placebo effects and observer bias. Finally, while Oura Ring data provide valuable trend information, wearable-based sleep staging does not replace polysomnography for definitive characterization of sleep architecture in pathological states (Svensson et al., 2024).

## 5 Implications for future research

Accordingly, this case does not advocate for a specific therapeutic regimen. Instead, it serves as a catalyst for structured investigation into immune contributions to refractory insomnia. Prospective cohort studies are needed to define the prevalence of histamine intolerance or mast cell activation features in post-viral insomnia populations. Controlled trials isolating the independent effects of H1/H2 blockade, dietary histamine restriction, and environmental trigger reduction are necessary to determine whether histaminergic modulation confers benefit beyond placebo. Integration of biochemical markers with gold-standard sleep measurements will be essential to validate or refute the proposed mast cell–sleep axis.

## 6 Patient perspective

“Before all of this began, I was an energetic, active person. I was golfing several times a week, out and about most of the day, and could easily complete high-intensity workouts several times per week. After the viral illness, everything changed. I went from thriving to barely functioning. I had crushing fatigue, was unable to sleep or nap, and felt as if my body had simply shut down. I tried to push through by forcing myself to exercise, hoping movement would help, but the fatigue was too profound.

I saw multiple specialists, underwent countless tests and a full cardiac work-up that all came back “normal,” and was offered antidepressants and stimulants. Deep down, I knew that was not what my body needed. It was frustrating and isolating to feel unseen inside a system that could not find an answer. After nearly a year of feeling as if I might just have to lie down and wait to die, the improvement after starting targeted therapy was almost immediate. Life-changing. For the first time since my illness, I slept deeply and woke rested. Within days, my energy, mood, and focus began to return, and I finally began to feel like myself again.”

## 7 Conclusion

This case describes an observed temporal association between histamine-targeted, environmental, and supportive interventions and significant objective and subjective improvement in a patient with post-viral refractory insomnia and dysautonomia. The observation supports the hypothesis that immune-mediated and histaminergic mechanisms may contribute to sleep disruption in a subset of patients with complex, treatment-resistant presentations. However, this single-case report does not establish causality or advocate for a specific therapeutic regimen. Instead, it highlights the need for systematic investigation into immune and neuroendocrine contributors to refractory insomnia using controlled study designs and validated biomarkers.

The patient provided written informed consent for publication of this case report and any accompanying data or images.

## Data Availability

The original contributions presented in the study are included in the article/supplementary material, further inquiries can be directed to the corresponding author.
